# Insights into the Vertical Stratification of Microbial Ecological Roles across the Deepest Seawater Column on Earth

**DOI:** 10.3390/microorganisms8091309

**Published:** 2020-08-27

**Authors:** Chun-Xu Xue, Jiwen Liu, David J. Lea-Smith, Gary Rowley, Heyu Lin, Yanfen Zheng, Xiao-Yu Zhu, Jinchang Liang, Waqar Ahmad, Jonathan D. Todd, Xiao-Hua Zhang

**Affiliations:** 1 College of Marine Life Sciences, Ocean University of China, Qingdao 266003, China; xuechunxu@outlook.com (C.-X.X.); liujiwen@ouc.edu.cn (J.L.); zhengyf90@126.com (Y.Z.); xiaoyuzhu321@126.com (X.-Y.Z.); liangjinchangluyao@163.com (J.L.); waqaryousufxai@outlook.com (W.A.); 2 Institute of Evolution & Marine Biodiversity, Ocean University of China, Qingdao 266003, China; 3 Laboratory for Marine Ecology and Environmental Science, Qingdao National Laboratory for Marine Science and Technology, Qingdao 266071, China; 4 School of Biological Sciences, University of East Anglia, Norwich Research Park, Norwich NR4 7TJ, UK; D.Lea-Smith@uea.ac.uk (D.J.L.-S.); G.Rowley@uea.ac.uk (G.R.); Jonathan.Todd@uea.ac.uk (J.D.T.); 5 School of Earth Sciences, University of Melbourne, Parkville, VIC 3010, Australia; linheyu@outlook.com; 6 Frontiers Science Center for Deep Ocean Multispheres and Earth System, Ocean University of China, Qingdao 266100, China

**Keywords:** Mariana Trench, hadal water, metagenomics, microbial community, function, metagenome-assembled genomes

## Abstract

The Earth’s oceans are a huge body of water with physicochemical properties and microbial community profiles that change with depth, which in turn influences their biogeochemical cycling potential. The differences between microbial communities and their functional potential in surface to hadopelagic water samples are only beginning to be explored. Here, we used metagenomics to investigate the microbial communities and their potential to drive biogeochemical cycling in seven different water layers down the vertical profile of the Challenger Deep (0–10,500 m) in the Mariana Trench, the deepest natural point in the Earth’s oceans. We recovered 726 metagenome-assembled genomes (MAGs) affiliated to 27 phyla. Overall, biodiversity increased in line with increased depth. In addition, the genome size of MAGs at ≥4000 m layers was slightly larger compared to those at 0–2000 m. As expected, surface waters were the main source of primary production, predominantly from Cyanobacteria. Intriguingly, microbes conducting an unusual form of nitrogen metabolism were identified in the deepest waters (>10,000 m), as demonstrated by an enrichment of genes encoding proteins involved in dissimilatory nitrate to ammonia conversion (DNRA), nitrogen fixation and urea transport. These likely facilitate the survival of ammonia-oxidizing archaea α lineage, which are typically present in environments with a high ammonia concentration. In addition, the microbial potential for oxidative phosphorylation and the glyoxylate shunt was enhanced in >10,000 m waters. This study provides novel insights into how microbial communities and their genetic potential for biogeochemical cycling differs through the Challenger deep water column, and into the unique adaptive lifestyle of microbes in the Earth’s deepest seawater.

## 1. Introduction

With an average depth of 3688 m, interspersed with trench systems that reach depths of almost 11,000 m, the Earth’s oceans are a huge ecosystem encompassing a broad range of life adapted to its varying nutrient and environmental conditions. The vertical profile of oceans is stratified and categorized into five depth zones: epipelagic (0–200 m), mesopelagic (200–1000 m), bathypelagic (1000–4000 m), abyssopelagic (4000–6000 m) and hadopelagic (>6000 m), that are distinguished by distinct physicochemical properties, including differences in light, pressure, temperature and nutrients. These differences affect the composition and biochemical properties of the microbial communities inhabiting these vertical zones, including their roles in biogeochemical cycling [1,2,3]. Characterizing the ecological roles of microorganisms across the whole water column is therefore critical for understanding a range of marine processes including nutrient cycling and the deposition of organic matter in ocean sediments. However, our knowledge of ocean microbial ecology is biased towards shallow depths, notably the epipelagic zone, and is limited for deeper zones, specifically the mesopelagic and lower zones.

The epipelagic zone is the uppermost layer of seawater exposed to solar radiation. Access to light in this zone drives primary production in plants, cyanobacteria, algae and organisms encoding microbial rhodopsins [4,5]. At depths ranging from the mesopelagic to the abyssopelagic zones, microbial communities are thought to largely subsist on organic matter derived from photosynthetic organisms in the form of sinking particulate organic matter (POM), as well as the flux of semi-labile dissolved organic carbon [6]. Another carbon source in aphotic zones is produced by chemolithoautophic microorganisms, notably *Thaumarchaeota*, via an energy efficient variant of the 3-hydroxypropionate/4-hydroxybutyrate cycle [7]. *Thaumarchaeota* populations are abundant and key primary producers in mesopelagic to abyssopelagic waters [1,8,9,10,11]. Further insights into carbon transformation and other microbial-driven biogeochemical processes in aphotic zones have been revealed by recent metagenome sequencing studies. A survey of the global ocean microbiome identified a higher fraction of novel genes in the mesopelagic zone compared to the epipelagic zone, suggesting that organisms may need to adapt to a wider range of ecological niches at these depths [12]. Moreover, Salazar et al. found that half of the prokaryotic operational taxonomic units (OTUs) within the bathypelagic zone belonged to previously unknown taxa, suggesting that our understanding of the microbial processes occurring in this region is limited [13]. In the deepest hadopelagic zone, which is almost exclusively composed of trenches [14], significantly different populations dominated by heterotrophic bacteria have been identified [1,2,11].

The Mariana Trench is located in the western Pacific Ocean and includes the Challenger Deep, the deepest known natural trench in the world (~11,000 m). Several studies have explored the functional potential of microbes in the Mariana Trench (>10,000 m), revealing a population with a high proportion of hydrocarbon degrading bacteria [2,15]. However, these studies were limited to a specific functional profile or a small range of depths. Much less is known of the variation in the genetic potential of microbial populations that participate in biogeochemical cycling and how this correlates to community composition down full-scale vertical profiles from the surface to the deepest ocean (0–10,500 m). Recent studies have highlighted a clear shift in pelagic communities and functions during the transition from abyssal to hadal zones, especially hydrocarbon-degrading genes enriched near the trench bottom (10,400 m and 10,500 m) [1,2,16]. It is possible, based on trench topography and subduction-linked physical and chemical features, that the abundance of these heterotrophic microbes is due to the metabolism of locally recycled organic carbon and not POM derived from photosynthesis. Thus, the Mariana Trench provides a unique case study to investigate the relationship between water depth and microbial community diversity along with their functional potential. 

This study used metagenomics to reveal the variation existing in microbial communities and their potential to participate in different biogeochemical cycles within seven different water layers (water depths: 0, 2000, 4000, 8000, 9600, 10,400 and 10,500 m) of the Challenger Deep in the Mariana Trench. In addition, we reconstructed 726 metagenome-assembled genomes (MAGs) belonging to 27 phyla (10 uncultured) to reveal the potential microbial interactions crucial to ecosystem function. Our results revealed that in the deepest waters (>10,000 m), genes encoding enzymes involved in dissimilatory nitrate to ammonia conversion (DNRA), nitrogen fixation and urea transport were enriched, possibly providing additional ammonia for ammonia-oxidizing archaea (AOA) of the alpha lineage which are dependent on high ammonia concentrations. Furthermore, at >10,000 m, a high relative abundance of genes encoding enzymes involved in oxidative phosphorylation and the glyoxylate shunt was observed, suggesting enhancement of these microbial activities.

## 2. Materials and Methods

### 2.1. Sampling and Sequencing

Seawater samples were collected by Niskin bottles on board of the R/V Dong Fang Hong 2 at the Mariana Trench site 11.20° N, 142.19° E on September 2016, as part of our previous study [2], and at site 11.38° N, 142.41° E on March 2017, utilizing the same sampling methods. In total, 16 samples from seven different water depths (0, 2000, 4000, 8000, 9600, 10,400 and 10,500 m) were acquired. Seawater samples were filtered sequentially through 3 μm (TSTP, 142 mm, Millipore, Burlington, MA, USA) and 0.22 μm (GTTP, 142 mm, Millipore, MA, USA) polycarbonate membranes. Here, we define materials that are unable to pass through the 3 μm pore-size filter as particulate organic matter (POM) reservoirs. Materials that can pass through 3 μm filters are defined as dissolved organic matter (DOM) reservoirs. Hence, the microbial communities being collected on the 3-μm and 0.22-μm filters were designated as particle-associated and free-living fractions, respectively. The filters with microorganisms were stored at −80 °C prior to processing for sequencing. After DNA extraction, metagenomic sequencing was performed on two fraction-sized (0.22 μm and 3 μm filters) pools of microorganisms from these 16 samples.

### 2.2. Assembly and Binning

The metaWRAP-Read_qc module [17] was used to trim the raw sequence reads, remove human contamination, and produce quality reports for each of the sequenced samples. After filtering, the clean reads from each sample were assembled separately using MegaHit version 1.1.2 [18] due to the very large reads of which data ranged in size from 14.29 to 18.68 Gb. Three metagenomics binning software, MaxBin2 version 2.2.4 [19], metaBAT2 version 2.12.1 [20], and CONCOCT version 0.4.0 [21], were then used to bin assembly each sample separately in the metaWRAP-Binning module. Subsequently, the three final bin sets produced were consolidated into a single and more robust bin set with the minimum completion (-c 50) and maximum contamination (-x 10) parameters using the Bin_refinement module in metaWRAP. The completion and contamination of each bin was evaluated by CheckM version 1.0.7 [22]. Next, we used the Reassemble_bins module to improve bins with reassembly. Finally, 87 MAGs (MAG 1–87) at 0 m, 77 MAGs (MAG 88–164) at 2000 m, 224 MAGs (MAG 165–388) at 4000 m, 122 MAGs (MAG 389–510) at 8000 m, 71 MAGs (MAG 511–581) at 9600 m and 145 MAGs (MAG 581–726) at >10,000 m were assembled. The approximate taxonomy of each bin was obtained using CheckM. Salmon version 1.7 tool [23] was used to estimate the abundance of each scaffold in each sample, and the average MAG abundances. The abundances referred to as “genome copies per million reads” were standardized to the individual sample size (Table S4).

### 2.3. Relative Abundance, Functional Characterization and Metabolic Analyses

Gene prediction for individual MAG genomes and metagenome sequences was performed using Prodigal version 2.6.3 [24] with default settings (“-p meta” for metagenome sequences; without parameter “-p meta” for individual MAG genomes). Genes in all 16 metagenomes samples were clustered to generate the non-redundant gene set using CD-Hit [25] at 95% identity and 90% coverage. To determine the relative abundance of each gene, we mapped the genes from the non-redundant gene set against the high-quality reads of each individual metagenome (generating 16 sam files) using BWA-MEM (Li 2013) (bwa v0.7.15-r1140, MA, USA, using default setting). We used the unsorted sam files as input in pileup.sh (bbmap-38.22-0) [26] to determine the average coverage of each gene. The relative abundance of each gene in the non-redundant gene set was obtained through dividing the average coverage of each gene by the sum of the average coverage of all genes in each metagenome. 

Predicted genes were further characterized using KOALA (KEGG Orthology and Links Annotation) webserver using GhostKOALA panel (used parameters: genus_prokaryotes + family_eukaryotes) [27]. Based on these KO assignments, the level of completeness of specific pathways/genes in various carbon, nitrogen, and sulfur metabolic processes in individual genomes was assessed using the script KEGG-decoder.py (www.github.com/bjtully/BioData/tree/master/KEGGDecoder, Los Angeles, CA, USA). To assess and compare the relative abundances of specific pathways/genes in different metagenomes samples, we built an in-house DiTing software (https://github.com/xuechunxu/DiTing, Version 0.5, Qingdao, Shandong, China). This software uses unbiased specific formula for each pathway to estimate the relative abundance of each pathway. 

To study specific metabolic characteristics, several databases were used for reference. The MEROPS database [28] was used for the identification of peptidase genes. The amino acid sequences of genes were subjected to BLASTp searches (Version 2.5.0, Maryland, USA) against the gene databases as described above to match the optimal genes with a maximum e-value of 10^−5^, minimum identity of 30%, and minimum query coverage of 50%. Furthermore, we annotated CAZymes using dbCAN2 software [29], which integrates three tools (HMMER, DIAMOND and Hotpep). CAZymes were only confirmed when the two searches yielded positive results.

The differences in these genes/pathways among different water depths were examined using the Wilcoxon test. The bubble diagram was generated by TBtools software v0.6662 [30], and the heatmap was generated by pheatmap package in the R software (https://www.r-project.org/, Auckland, New Zealand).

### 2.4. Phylogenetic Analyses of the Reconstructed Genomes, AmoA Proteins and AOA Genomes

GTDB-Tk v0.1.3 [31] was employed to address an issue with MAGs assignments based on the placement of genomes in the reference tree. In total, 120 bacterial or 122 archaeal marker genes of each MAG genome were used for phylogenetic inference. We inferred maximum likelihood (ML) phylogenomic trees of MAGs with IQ-TREE version 1.6.1 [32] under the LG+R10 and LG+R4 model. For better visualization, the trees were beautified using the Interactive Tree of Life (iTOL) webtool [33].

Reference AmoA protein sequences were downloaded from the NCBI database. AmoA protein sequences in *Thaumarchaeota* MAGs and reference sequences were first aligned using MAFFT [34] and then adjusted with TrimAl v1.2 software [35]. The AmoA tree was inferred based on the ML algorithm using IQ-TREE version 1.6.1 [32]. In addition, we inferred maximum likelihood phylogenomic trees of AOA with IQ-TREE version 1.6.1 [32], based on a multiple sequence alignment of 122 marker proteins obtained using GTDB-Tk v0.1.3 [31]. We downloaded the AOA reference genomes from the BIGD database according to Wang Y et al. [15].

### 2.5. Data Availability

Sequence data for metagenomes obtained from the September 2016 and March 2017 cruises have been deposited in NCBI as part of our previous studies [2] under BioProject numbers of PRJNA412741 and PRJNA541485, respectively. The 726 MAGs have been deposited under the BioProject number of PRJNA563531.

## 3. Results 

### 3.1. Phylogenetic Diversity through the Water Column in the Mariana Trench

**Figure 1:**
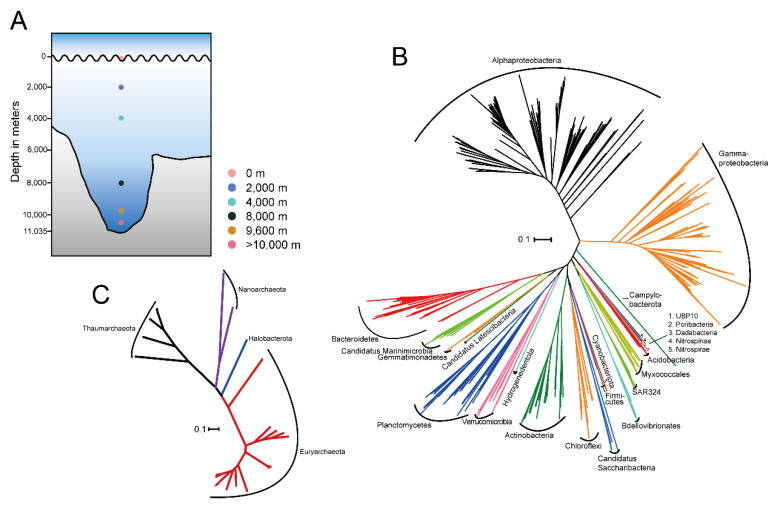
Sampling sketch and phylogenetic inference of metagenome-assembled genomes (MAGs). (**A**) Sampling sketch across a seawater column in the Mariana Trench. Phylogenetic inference of 693 bacterial MAGs (**B**) and 33 archaeal MAGs (**C**) based on 120 and 122 single-copy, protein-coding marker genes, respectively. All MAGs were assigned to 28 clusters represented by different branch colors. The scale bar represents 0.1 substitutions per site.

To examine the genetic potential of microbial communities across the water column, we performed metagenomic sequencing from 16 samples ranging from depths of 0 to 10,500 m (Figure 1A) in the Challenger Deep. After sequence assembly, we reconstructed 726 draft genomes via tetranucleotide and coverage binning. These metagenome-assembled genomes (MAGs) represented 254 high-quality MAGs (>90% complete and <5% contaminated) and 472 medium-quality MAGs (≥50% and <10% contaminated) [36] (Table S1). In general, more genomes were recovered as the depth increased, from an average of 29 MAGs per sample at 0 m to 71 at 9600 m, suggesting greater biodiversity in the deep-sea environment in general. However, only 36.25 MAGs per sample on average were recovered in the >10,000 m samples. An increase in genomic diversity in deep seawater may correlate with a higher capacity of organisms in this environment to degrade or use different substrate types [37,38,39]. The genome size of MAGs at ≥4000 m layers was slightly larger compared to those from upper layers (0 m and 2000 m) (Figure S1A). The GC content of MAGs was comparable at all depths (Figure S1B).

A phylogenetic analysis of the single-copy, protein-coding marker genes (120 for bacteria and 122 for archaea) revealed 693 bacterial and 33 archaeal genomes, which represented 17 cultured and 10 uncultured candidate phyla according to the GTDB database (Figure 1B,C, Table S1) [31]. Similarly, taxonomic classification performed on unassembled reads using Kraken 2 showed that more than 90% of all taxonomically assigned reads matched bacteria, followed by archaea (ranged 0.16–1.66%), eukaryotes (0.62–4.21%) and viruses (0.08–3.67%) (Table S2). The community compositions derived from MAGs and metagenomic reads were similar in general, with *Alphaproteobacteria*, *Gammproteobacteria* and *Bacteroidetes* being dominant across all depths (Figure S2). However, archaea were less frequently detected in metagenomic reads (0.16–1.66%) than in MAG taxonomy (2.23–12.41%) (Table S2), which may be due to the smaller genome size of archaea [40]. Despite the slight variations between the MAG- and read-based taxonomy, the genomes accurately reflected the community as a whole and shed light on ecologically significant variations in community structure and/or metabolic potential that occur even at the finite levels of diversity, such as between ecotypes, clonal lineages or within species [39,41,42].

### 3.2. Phototrophy and Carbon Fixation

**Figure 2:**
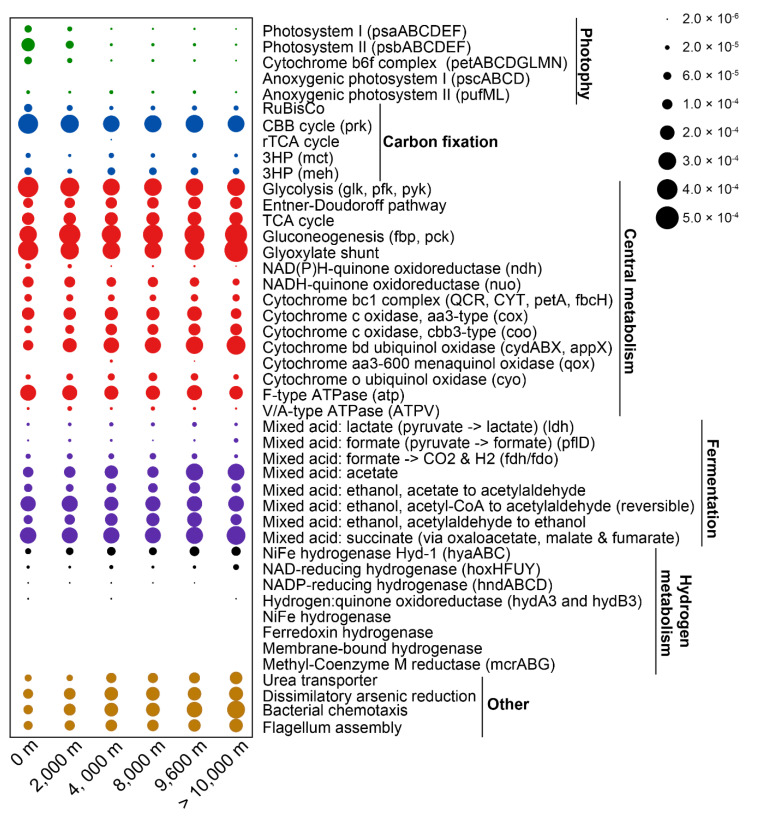
Distribution of major functional processes across different water depths in the Mariana Trench. The *X*-axis indicates the different water layer samples were derived from. The *y*-axis indicates the major selected functional processes involved in oxidative phosphorylation, carbon fixation, carbon degradation, hydrogen redox and other specific pathways. Distribution of these functional processes in each sample can be found in Figure S3.

**Figure 3:**
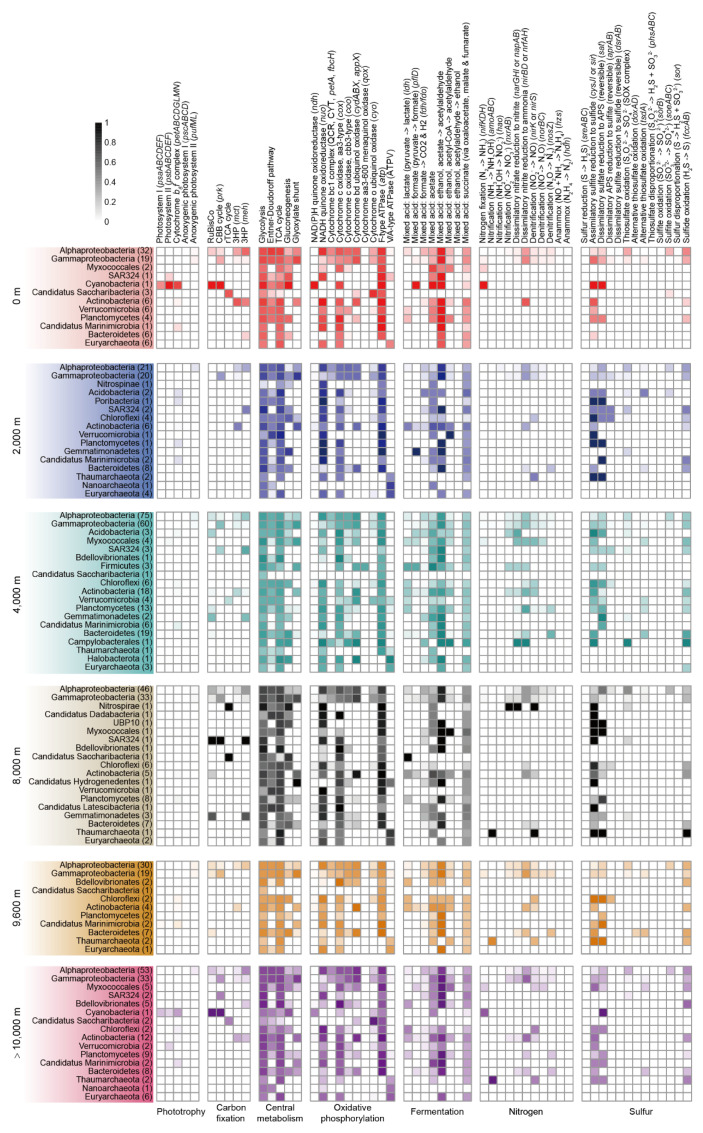
Selected processes detected across phylogenetic clusters inhabiting different depths. Average completeness of selected processes corresponding to Figure 2 for each phylogenetic cluster. Number in brackets: number of genomes belonging to individual phylogenetic clusters at each depth. A complete list of processes in 726 MAGs can be found in Table S3.

As expected, many subunits from the complexes required for oxygenic photosynthesis, including photosystem I (*psaABCDEF*), photosystem II (*psbABCDEF*) and cytochrome *b*_6_*f* (*petABCDGLMN*), were most abundant in surface seawater samples (Figure 2). Photosystem I and II, and the cytochrome *b*_6_*f* complex, were mostly affiliated to the cyanobacterium *Crocosphaera watsonii* WH8501, an abundant diazotrophic marine cyanobacterium found in surface waters (Figure 3, Table S3) [43]. Another *C. watsonii* WH8501 MAG was reconstructed from the >10,000 m metagenomes, suggesting that biomass from the surface sank to this depth. We next assessed the MAGs for photoheterotrophy in organisms undertaking anoxygenic photosynthesis by searching for subunits of the reaction center complexes (*pscABCD* and *pufML* genes). The *pscABCD* genes encoding subunits in the type I reaction center were absent in all MAGs. The *pufML* genes encoding subunits in the type II reaction center were detected at similar levels across the depth profile in both metagenome assemblies and MAGs (Figure 2 and Figure 3). As expected, aerobic anoxygenic photosynthesis was predominantly associated with Proteobacteria [44]. This suggests either that some of the biomass from photoheterotrophs sinks from surface waters or these organisms migrate throughout the seawater column. To identify potential differences in primary production throughout the water column, we compared marker genes for carbon fixation. The potential for Calvin–Benson–Bassham (CBB) cycling was originally assessed via the presence of the marker genes for Group I/II ribulose-1,5-bisphosphate carboxylase/oxygenase (RuBisCO) and phosphoribulokinase (*prk*). RuBisCO and *prk* distribution were similar across the water column with the highest abundance in the surface seawater (especially in the free-living fraction) (Figure S3). The majority of RuBisCO and *prk* genes were found in *Alphaproteobacteria* and *Cyanobacteria*. These *Alphaproteobacteria* also contained the genes *soeABC* or *fccAB*, encoding subunits of the SOX complex involved in reduced sulfur oxidation, indicating they are likely chemoautotrophic. The *Gammaproteobacteria*, especially *Alteromonadales* MAGs, had *prk* genes, but lacked RuBisCO. In these genomes, the *prk* genes were consistently adjacent to phosphodiesterase, indicating the *prk* may be involved in pentose phosphate metabolism. Decoupling of *prk* and RuBisCO undermines the utility of *prkB* as a marker gene for the CBB cycle within the *Alteromonadales* group.

The reverse tricarboxylic acid (rTCA) cycle, used by some anaerobic and microaerobic bacteria for carbon fixation, was investigated using the marker genes ATP citrate lyase (*aclAB*) and citryl-CoA synthetase (*ccsAB*), both of which were in low abundance across the depth profile (Figure 2). The rTCA cycle was linked to six MAGs, including four *Candidatus Saccharibacteria*, one *Nitrospirae* and one *Verrucomicrobia* (Figure 3, Table S3). To the best of our knowledge, this is the first report of an rTCA cycle in *Saccharibacteria*, a candidate phylum which survives in an epibiotic parasitic lifestyle due to its extreme auxotrophy [45]. In the *Nitrospirae* MAG, the nitrite-oxidation gene (*nxrAB*) is present alongside the rTCA cycle, which is consistent with this species being an anaerobic chemoautotroph [46]. Wood–Ljungdahl-mediated anaerobic carbon fixation appeared to be absent in the analyzed meta-/genomes based on the absence of the marker genes carbon monoxide dehydrogenase (*cooS*), acetyl-CoA decarbonylase/synthase (*cdhAB*), acetyl-CoA synthase (*acsB*) and 5-methyltetrahydrofolate corrinoid/iron sulfur protein methyltransferase (*acsE*) (Table S3). Two marker genes, 2-methylfumaryl-CoA isomerase (*mct*) and 3-methylfumaryl-CoA hydratase (*meh*), encoding proteins required for the 3-hydroxypropionate bi-cycle (3HP cycle), were mostly restricted to *Alpha*- and *Gamma*-*proteobacteria* and *Actinobacteria*. Over half (69.2%) of these MAG genomes also possessed the SOX complex genes *soeABC* or *fccAB*, for reduced sulfur oxidation, providing some evidence for chemoautotrophy (Table S3). A higher proportion (18.4%; 16/87) of surface seawater derived MAG genomes encoded enzymes involved in the 3HP cycle than in deep sea-derived genomes (8.6%; 55/639).

Overall, photoautotrophy was primarily localized to surface seawater samples. Potential chemoautotrophic MAGs (the coupling of carbon fixation and reduced compound oxidation) were mostly linked to *Alphaproteobacteria* (53/74), followed by *Gammaproteobacteria* (11/74), *Actinobacteria* (9/74) and *Nitrospirae* (1/74). Chemoautotrophic bacteria were more prevalent in surface samples than deep-sea (14.9% (13/87) vs. 9.5% (61/639), respectively). These results suggest that in surface waters, photoautotrophy and chemoautotrophy drives primary production, while in the deep sea (≥2000 m), this is predominantly derived via chemoautotrophic processes. The data here also suggest bacteria and not archaea are the main primary producers.

### 3.3. Complex Carbon Degradation

**Figure 4:**
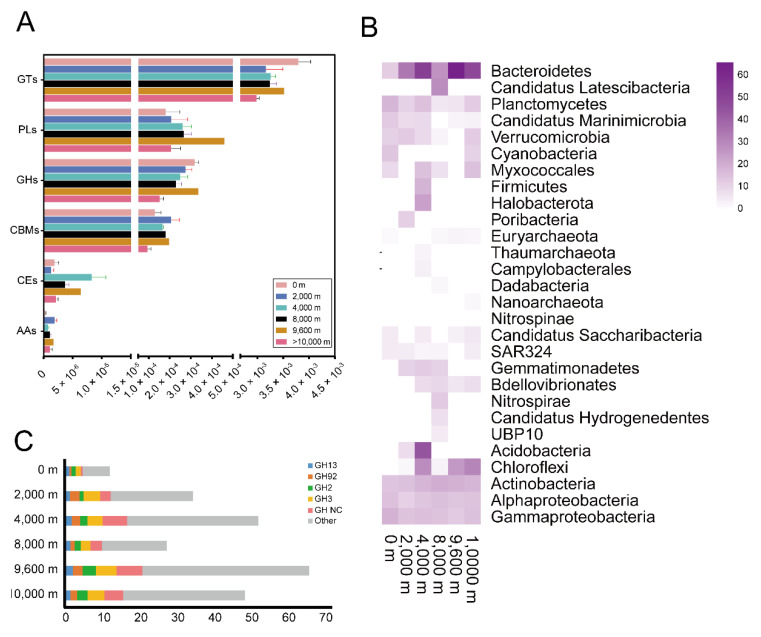
The relative abundances of genes involved in the microbial degradation pathways of carbohydrates across different water depths in the Mariana Trench (**A**). GTs, glycosyltransferases; PLs, polysaccharide lyases; GHs, glycoside hydrolases; CBMs, carbohydrate-binding modules; CEs, carbohydrate esterases; AAs, auxiliary activities. The number of glycoside hydrolases in the MAGs across different water depths in the Mariana Trench (**B**). The number of the main glycoside hydrolase from *Bacteroidetes* across different water depths in the Mariana Trench (**C**).

Carbohydrates and peptides in seawater can be degraded and metabolized by microorganisms through carbohydrate-active enzymes (CAZymes) and peptidases. CAZymes are involved in polysaccharide production and degradation, providing an advantage for heterotrophs in utilizing related organic matter. Glycosyltransferases (GTs), catalyzing the formation of the glycosidic linkage to form a glycoside, were highly abundant CAZymes in all samples, most notably at the surface (Wilcoxon test, *p* < 0.05) (Figure 4A, Figure S4). Glycoside hydrolases (GHs) and Polysaccharide lyases (PLs) were similar in relative abundance, but were in highest abundance at 9600 m.

By searching for GHs in MAG genomes, we found that bacteria encoded for a broader repertoire of CAZymes than archaea (Figure 4B, Table S5). Most CAZymes were assigned to *Bacteroidetes* (n = 40), followed by *Actinobacteria* (n = 18), *Gammaproteobacteria* (n = 15), *Chloroflexi* (n = 15), *Alphaproteobacteria* (n = 14) and *Planctomycetes* (n = 11). *Bacteroidetes* MAGs derived from the deep sea (≥2000 m) showed a substantially higher number of GHs compared to surface-derived *Bacteroidetes* MAGs (average 46 and 12 GHs for ≥2000 m and 0 m, respectively) and included a large number of glycoside hydrolases not yet assigned to a family (Figure 4C). These data suggest that certain microorganisms, such as *Bacteroidetes*, might have acquired novel GH genes to degrade carbohydrates specific to the deep sea.

Peptidases were equally distributed across the depth profile. The free-living microorganisms showed a higher relative abundance of peptidases than the particle-attached counterparts (Figure S5A). The peptidase:CAZyme ratios were correspondingly higher in free-living microorganisms than those associated with particles (Figure S5B). This is consistent with previous studies demonstrating that free-living *Flavobacteriia* with small genomes have a higher peptidase:CAZyme ratio, compared to particle-associated *Flavobacteriia* with large genomes, since these organisms preferentially utilize polysaccharides [47,48,49,50]. 

Genes encoding enzymes involved in the degradation of aliphatic and aromatic hydrocarbons were investigated (Figure S6). Alkane degradation genes (*alkB* and *almA*) significantly increased in the near bottom waters (>10,000 m) (Wilcoxon test, *p* < 0.05) (Figure S6C), as reported by Liu et al. (2019). The relative abundance of *almA* was higher than that of *alkB* across all depths (Figure S6B). In comparison, genes encoding enzymes involved in degradation of aromatic hydrocarbons, including toluene, xylene, benzene, benzoate, naphthalene, salicylate, phthalate, and catechols, were enriched in the 4000–9600 m samples (Wilcoxon test, *p* < 0.05) (Figure S6D), with the most abundant genes being involved in catechol meta-cleavage, followed by catechol ortho-cleavage and benzene degradation (Figure S6B). Microorganisms in >10,000 m waters display a higher ratio of genes encoding alkane degradation rather than aromatic degradation, compared to those isolated from 0 to 9600 m (Wilcoxon test, *p* < 0.05) (Figure S6E). Overall, these data suggest that microorganisms in >10,000 m waters utilize alkanes more than aromatics, with the opposite occurring in 0–9600 m waters.

### 3.4. Central Metabolism

The central metabolic pathways for degradation of substrates produced by CAZymes and peptidases include glycolysis, the Entner−Doudoroff pathway, the TCA cycle, gluconeogenesis and anaplerotic reactions. We first examined the distribution of genes encoding enzymes specifically involved in glycolysis and gluconeogenesis. Genes encoding enzymes only involved in glycolysis (*glk*, *pfk*, *pyk*) showed the highest abundance in the free-living fraction in surface waters (Wilcoxon test, *p* < 0.05) (Figure S3), while the least abundance was observed in >10,000 m waters (Wilcoxon test, *p* < 0.05) (Figure 2). Inversely, genes encoding enzymes only involved in gluconeogenesis (*fpb, pck*) showed the least abundance in surface waters (Wilcoxon test, *p* < 0.05), but were most abundant in the free-living 10,000 m fraction (Wilcoxon test, *p* < 0.05) (Figure 2). The Entner−Doudoroff and TCA pathways were equally distributed across all depths. The glyoxylate shunt, which acts as an alternative to the TCA cycle, providing biosynthetic intermediates and bypassing the decarboxylation steps, was enriched at >10,000 m (Figure 2). This may be an adaptive strategy for bacteria at >10,000 m layers to reduce carbon demand. The glyoxylate shunt was mainly found in *alpha*- and *gamma*-*proteobacteria* and other clusters including *Actinobacteria* and *Bacteroidetes* (Figure 3). 

Oxidative phosphorylation is an important part of central metabolism for aerobic microorganisms. NAD(P)H dehydrogenase (*ndh*) was strictly limited to photoautotrophic *Cyanobacteria*, while NADH dehydrogenase (Complex I) (*nuo*) was distributed widely in all other MAGs. The relative abundance of both dehydrogenases tended to decrease with sampling depth (Figure 2). Cytochrome *bc*_1_ complex (Complex III) (QCR, CYT, *petA*, *fbcH*) was identified in MAGs of *alpha*- and *gamma*- *proteobacteria* at all depths and *Campylobacterales* from 4000 m. The *aa*_3_-type cytochrome c oxidase, which has low affinity for O_2_, was least abundant in the deepest layers (>10,000 m), while the *cbb*_3_-type cytochrome c oxidase, with high affinity for O_2_, was most abundant at ≥4000 m [51]. The *aa*_3_-type cytochromes were found in over 90% of total MAGs and at all depths. Surprisingly, more *cbb*_3_-type cytochrome containing MAGs were reconstructed from ≥4000 m than 0 m and 2000 m (62.8% vs. 46.3%). These *cbb*_3_-type MAGs also contained *aa*_3_-type cytochromes, suggesting adaptation of the species encoding both types to a range of oxygen concentrations. Similar to the *cbb*_3_-type, the relative abundance of cytochrome *bd* ubiquinol oxidase (*cydABX*, *appX*), which also displays high oxygen affinity, gradually increased at lower depth and was significantly enriched at >10,000 m (Wilcoxon test, *p* < 0.05) (Figure 2). The quinol-based cytochrome oxidases, cytochrome *aa*_3_-600 menaquinol oxidase (*qox*) and cytochrome o ubiquinol oxidase (*cyo*), were not abundant at any depth (Figure 2). Cytochrome *aa*_3_-600 menaquinol oxidases were only found in two *Firmicutes* MAGs derived from 4000 m (Figure 3). Cytochrome o ubiquinol oxidases were prevalent in more diverse MAGs, including *alpha*- and *gamma*-*proteobacteria*, *Verrucomicrobia* and *Candidatus Saccharibacteria*. ATPase (Complex V) synthesizes ATP, with the main F-type (*atp*) being most abundant in the free-living fraction in surface waters and widespread in most bacterial MAGs (Figure 2, Figure 3 and Figure S3). As expected, the V/A-type ATPase (ATPV) was present in archaea.

### 3.5. Fermentative Metabolism

Fermentation is of central importance in anaerobic microorganisms. Lactate metabolism was assessed by the presence of the *L*-lactate dehydrogenase encoding gene (*ldh*), which converts pyruvate to lactate. Seventy-five bacterial MAGs assigned to 11 clusters from all depths possessed *ldh* genes, while these genes were absent in archaea (Figure 3, Table S3). The relative abundance of *ldh* gene was low and roughly equal at all depths (Figure 2). Formate metabolism was assessed by the presence of formate C-acetyltransferase (*pflD*), which catalyzes formate formation from pyruvate, and formate dehydrogenase (*fdh*/*fdo*), catalyzing formate oxidation to CO_2_ and H_2_. The *pflD* gene was distributed in 24 MAGs from eight phyla. The *fdh*/*fdo* gene was identified in 264 MAGs. Acetate can be produced from pyruvate (*poxB*), acetyl-P (*poxL*, *acyP*), lactate (E1.13.12.4) and acetyl-CoA (K04020, K13788 and K00625). The potential for acetate metabolism was prevalent in over half of all MAGs (n = 382). Ethanol metabolism was detected by searching for the genes encoding aldehyde dehydrogenase (K00128, K14085, K00149, K00129 and K00138) and acetaldehyde dehydrogenase (E1.2.1.10), catalyzing acetate and acetyl-CoA conversion to acetylaldehyde, respectively, and alcohol dehydrogenase (*adh*), which catalyzes the conversion of acetylaldehyde to ethanol. The relative abundances of these genes did not differ significantly throughout the depth profile (Wilcoxon test, *p* 0.05) (Figure 2). Both aldehyde dehydrogenase and acetaldehyde dehydrogenase were widespread in the retrieved MAGs. *Adh* was only detected in two *Gammaproteobacterial* MAGs belonging to the genus *Methylophaga* (Figure 3).

Various types of hydrogenases, that conduct H_2_ metabolism, were analyzed. Four kinds of [NiFe]-hydrogenases were identified in the metagenomes and MAGs, including the oxygen tolerant [NiFe]-hydrogenase Hyd-1 (*hyaABC*), NAD-reducing hydrogenase (*hoxHFUY*), NADP-reducing hydrogenase (*hndABCD*) and hydrogen:quinone oxidoreductase (*hydA3* and *hydB3*). *HyaABC* and *hoxHFUY* were distributed throughout the water column at low abundance. The other two hydrogenases were present in only a fraction of samples. These genes were mostly affiliated with *Alphaproteobacteria*, *Gammaproteobacteria*, *Actinobacteria, Bacteroidetes* and *Chloroflexi* (Table S3). These findings indicated a potentially low level of hydrogen metabolism in the Mariana Trench.

The key enzyme for methanogenesis, methyl-Coenzyme M reductase (*mcrABG*), was not detected in any metagenomes or MAGs. For methane oxidation, we identified the genes encoding the enzyme catalyzing methane to methanol, methane/ammonia monooxygenase (*amoABC*), in five *Thaumarchaeota* MAGs. These data suggest that methane metabolism is likely present at very low levels in the Mariana Trench.

### 3.6. Nitrogen Cycling

**Figure 5:**
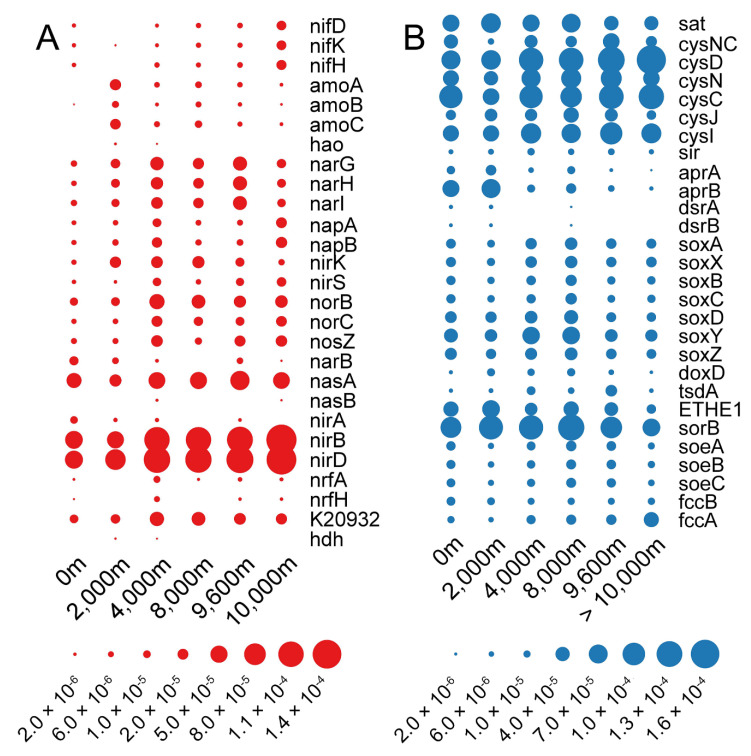
Relative abundance of genes involved in the microbial nitrogen (**A**) and sulfur (**B**) cycle in the six depths examined in the Mariana Trench. *NifDKH*, nitrogenase molybdenum-iron protein; *amoABC*, methane/ammonia monooxygenase; *hao*, hydroxylamine dehydrogenase; *narGHI*, nitrate reductase; *napAB*, nitrate reductase; *nirKS*, nitrite reductase; *norBC*, nitric oxide reductase; *nosZ*, nitrous oxide reductase; *narB*, ferredoxin nitrate reductase; *nasAB*, assimilatory nitrate reductase; *nirA*, ferredoxin-nitrite reductase; *nirB*, nitrite reductase (NADH) large subunit; *nirD*, nitrite reductase (NADH) small subunit; *nrfA*, nitrite reductase (cytochrome c-552); *nrfH*, cytochrome c nitrite reductase small subunit; K20932, hydrazine synthase subunit; hdh, hydrazine dehydrogenase. *sat*, sulfate adenylyltransferase; *cysNC*, bifunctional enzyme CysN/CysC; *cysD*, sulfate adenylyltransferase subunit 2; *cysN*, sulfate adenylyltransferase subunit 1; *cysJ*, sulfite reductase (NADPH) flavoprotein alpha-component; *cysI*, sulfite reductase (NADPH) hemoprotein beta-component; sir, sulfite reductase (ferredoxin); *aprAB*, adenylylsulfate reductase; *dsrAB*, dissimilatory sulfite reductase; *soxABCDXYZ*, thiosulfate oxidation by SOX complex; *doxD*, thiosulfate dehydrogenase (quinone) large subunit; *tsdA*, thiosulfate dehydrogenase; ETHE1, sulfur dioxygenase; *sorB*, sulfite dehydrogenase; *soeABC*, sulfite dehydrogenase; *fccAB*, sulfide dehydrogenase.

**Figure 6:**
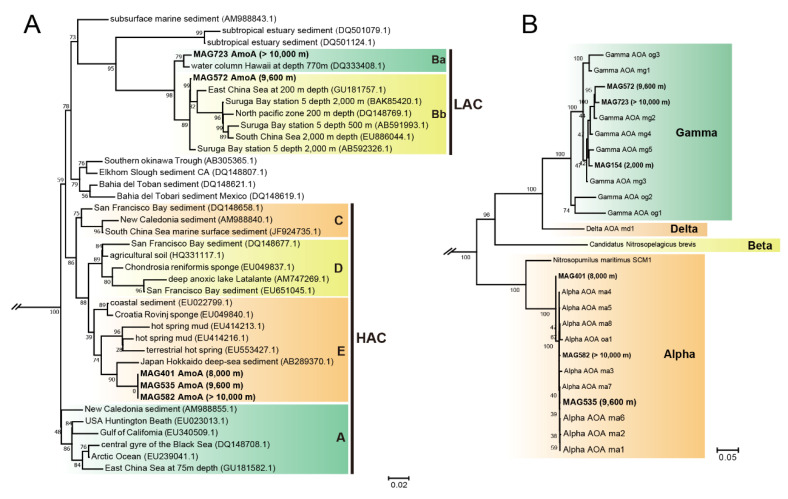
Phylogenetic tree of AmoA proteins and ammonia oxidation archaea. (**A**) AmoA proteins were used for maximum likelihood (ML) phylogenetic reconstruction with reference sequences from the NCBI database. LAC: low ammonia concentration; HAC: high ammonia concentration. The sequences in bold were extracted from MAGs of this study. (**B**) Phylogenetic inference of ammonia-oxidizing archaea based on 122 single-copy, protein-coding marker genes.

The known processes within the nitrogen cycle include nitrogen fixation, nitrification, denitrification, assimilation and anammox, through which atmospheric nitrogen (N_2_) is converted into biologically available forms. A complete set of nitrogen-fixing genes (*nifDKH*) converting dinitrogen into ammonia (NH_4_^+^) or related nitrogenous compounds were significantly enriched at >10,000 m waters (Wilcoxon test, *p* < 0.01) (Figure 5A). Nitrogen fixers had a narrow taxonomic distribution and *nifKDH* genes were only found in one *Cyanobacteria* MAG, three *Alphaproteobacteria* MAGs and six *Gammaproteobacteria* MAGs (Figure 3, Table S3). According to the distribution pattern of these taxa, *Cyanobacteria* are likely the major diazotrophs in surface waters, while *Alphaproteobacteria* and *Gammaproteobacteria* dominated this process in deep waters (≥2000 m). Nitrification is made up of two reactions, i.e., ammonia oxidation (NH_3_ → NH_2_OH → NO_2_^−^) and nitrite oxidation (NO_2_^−^ → NO_3_^−^). The ammonia oxidation pathway (*amoABC*) showed highest relative abundance in 2000 m waters and was strictly limited to *Thaumarchaeota* MAGs. Among the five hadal water-derived ammonia-oxidizing archaeal (AOA) *Thaumarchaeota* MAGs, three (MAG401, 535, 582 retrieved from 8000, 9600, and >10,000 m, respectively) were clustered with the α lineage and possessed the high ammonia concentration (HAC)-*amoA* gene group E (Figure 6). The other two (MAG572, 723 retrieved from 9600 and >10,000 m, respectively) were affiliated with the γ lineage and possessed the low ammonia concentration (LAC)-*amoA* group Ba and Bb. *Hao*, encoding for the enzyme converting the ammonia-oxidation intermediate hydroxylamine to NO_2_^−^, was only detected in one *Alphaproteobacteria* MAG retrieved from 2000 m. For nitrite oxidation, genes *nxrAB*/*narGH* (nitrite oxidoreductase) were only identified in two MAGs (2/164, 1.22%) from 0 to 2000 m, which were *Alphaproteobacteria* and *Gammaproteobacteria*, respectively. At ≥4000 m, genes *nxrAB*/*narGH* were spread widely in 42 MAGs (42/562, 7.47%), including *Alpha-*, *Gammaproteobacteria*, *Acidobacteria*, *Firmicutes*, *Actinobacteria* and *Nitrospirae*, indicating *nxrAB*/*narGH* genes may be horizontally transferred in populations at >4000 m layers.

Denitrification contains four enzymatic steps for the serial reduction of NO_3_^−^ into N_2_, encoded by *nar*, *nir, nor* and *nos*, respectively, and leads to a net loss of N from the local environment. Overall, compared to the seven MAGs (7/87) from the surface, far more, 148 (148/639), were found in deep water (≥2000 m) derived MAGs possessing genes required for complete/partial denitrification. These were more phylogenetically diverse (Figure 3, Table S3), reflecting, as expected, increased metabolism of these alternative electron acceptors at ≥2000 m depths compared to the surface. Dissimilatory nitrate reduction to ammonia (DNRA), indicated by marker genes for nitrite reductase (*nirBD* or *nrfAH*), anaerobically oxidizes organic matter and reduces nitrate/nitrite to ammonium. In contrast to denitrification, DNRA recycles the bioavailable nitrogen for the local habitat (NO_2_^−^ → NH_4_^+^) [52] and showed the highest relative abundance among all genes for nitrogen cycling in each sample (Figure 5A). Denitrification and DNRA compete for available nitrate/nitrite in natural systems and the partitioning of these processes is impacted by a range of environmental factors, including oxygen levels, N-oxyanion and electron donor availability (C/N ratio), and metal cofactors required for functional enzymes. For example, DNRA may outcompete denitrification when nitrate is low but there is an excess of electron donors [53]. *NirBD* or *nrfAH* was found in 250 bacterial MAGs represented by *Alphaproteobacteria*, *Gammaproteobacteria*, *Actinobacteria*, *Planctomycetes* and *Bacteroidetes*, but not in archaea. Notably, the mean relative abundance of DNRA in the deepest layers (>10,000 m) was highest compared to the upper layers (Wilcoxon test, *p* < 0.01) (Figure 5A). 

### 3.7. Sulfur Cycling

Microorganisms can reduce sulfate in both an energy-consuming assimilatory pathway and an energy-producing dissimilatory pathway. Microorganisms use *PAPSS*, *sat* and *cysCNDH* genes that encode for enzymes that assimilate sulfate and reduce it to sulfite, and *cysJI* and *sir* genes that encode for enzymes that facilitate sulfite reduction to sulfide. Four of these genes (*cycD*, *cysN*, *cysC* and *cysI*) were significantly more abundant at ≥4000 m compared with 0 and 2000 m (Wilcoxon test, *p* < 0.05) (Figure 5B). Microbial *sat* and *aprAB* genes encode for enzymes involved in dissimilatory reduction of sulfate to sulfite, and *dsrAB* genes encode for enzymes involved in dissimilatory reduction of sulfite to sulfide. Marker genes *aprAB* and *dsrAB* were in higher abundance at 0 m and 2000 m compared to other levels (Wilcoxon test, *p* < 0.01). Marker genes for dissimilatory pathways were limited to *Alpha-*, *Gamma-proteobacteria,* SAR324 and *Chloroflexi*. These data suggested that microorganisms at 0 m and 2000 m are more inclined to use the dissimilatory pathway, while microorganisms at ≥4000 m are more inclined to use the assimilatory pathway.

The SOX (sulfur-oxidation) system (*soxABCDXYZ*) is a well-known thiosulfate oxidation pathway in many sulfur bacteria, which can oxidize thiosulfate to sulfate. Oxidation of inorganic sulfur compounds is linked with energy production via membrane-bound electron transport chains and may be coupled with carbon dioxide fixation [54,55]. *SoxABCDXYZ* genes were significantly more abundant at 4000 and 8000 m (Figure 5B). The complete thiosulfate oxidation pathway was identified in *Alphaproteobacteria*, *Gammaproteobacteria* and one *Campylobacterota* MAGs (Figure 3, Table S3). The other sulfur oxidation genes, *tsdA* and *doxAD*, encoding thiosulfate dehydrogenases, were also detected in relatively low abundance [56] (Figure 5B). Thiosulfate reductase (*phsABC*), required for thiosulfate disproportionation, and sulfur oxygenase (*sor*), required for sulfur disproportionation, were not found in any MAGs (Figure 3). Sulfite dehydrogenase (*soeABC*), involved in sulfite oxidation, showed a higher abundance at 8000 m and 9600 m, followed by 0 m and 4000 m (Figure 5B), and were mainly found in *Alphaproteobacteria* MAGs.

## 4. Discussion

**Figure 7:**
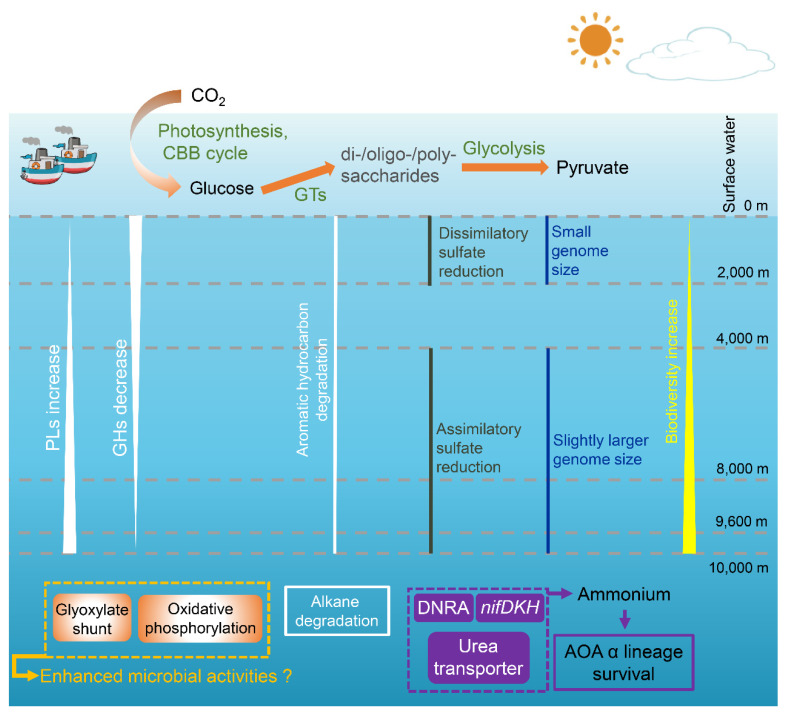
Selected processes throughout the water column. The biodiversity increased along the depths increased. The genome size of MAGs at ≥4000 m layers was slightly larger compared to those at 0–2000 m. Glycoside hydrolases (GHs) and Polysaccharide lyases (PLs) had inverse vertical variation patterns, showing a generally declining and increasing trend with depth, respectively. Microorganisms in >10,000 m waters utilize alkanes more than aromatics, with the opposite occurring in 0–9600 m waters. Microorganisms at 0–2000 m are more inclined to use the dissimilatory sulfate reduction pathway, while microorganisms at ≥4000 m are more inclined to use the assimilatory sulfate reduction pathway. In surface waters, the relative abundance of genes for photosynthesis and CBB cycle was enriched, and thus provides the main source of primary production. In addition, glycosyltransferases (GTs) occupied the highest proportion at the surface layer compared to other depths, indicative of a higher potential of carbohydrate biosynthesis in this environment. Glycolysis was most abundant in surface waters. The deepest waters (>10,000 m) are enriched for DNRA, nitrogen fixation and urea transport, which could provide an additional ammonia source for ammonia oxidation and survival of the α lineage of the AOA. Additionally, microbial activities may be enhanced at >10,000 m with a higher relative abundance of proteins involved in oxidative phosphorylation and the glyoxylate shunt.

In this study, we generated 16 metagenomes and 726 MAGs to assess the vertical variation of microbial community composition and metabolic potential across the deepest seawater column on Earth. Our results revealed that microbial communities and metabolic potential were different at each depth (Figure 7). Our findings suggest that hydrostatic pressure affects the evolution of a range of microbial metabolic functions, resulting in distinct populations at each depth. This study is the first to assess both the microbial communities and metabolic potential across the largest range of vertical depths and provides further insight into the microbial ecosystem in the cryptic deepest ocean on Earth. Similar to previous work on the enhanced biodiversity in deep-sea ecosystems, including ridges, deep-water coral reefs, cold seeps, brine pools, gas hydrates, fractures, and trenches [57,58,59,60], we demonstrated that compared to the surface, the deep-sea (≥2000 m) in the Mariana Trench contained enhanced biodiversity. The more extreme conditions in the deep sea, especially hydrostatic pressure, do not seem to limit microbial diversity. This is confirmed by the high diversity of culturable bacteria isolated from the deep-sea (>2000 m) in the Mariana Trench [61]. Notably, compared to 9600 m, the >10,000 m samples showed a decreased number of average MAGs. The >10,000 m layers were close to the surface sediment of the Mariana Trench, which may affect the community composition in terms of biodiversity and function.

Based on the functional potential of identified genes and the MAG results, we identified the dominant microbial metabolic processes and population dynamics at each depth. As expected, the relative abundance of genes for photosynthesis and the CBB cycle was enriched in surface waters. Genomic binning further indicated that the main photoautotrophic group coupling oxygenic photosynthesis and the CBB cycle were *Cyanobacteria*. In addition, glycosyltransferases (GTs) were found in the highest proportion in surface waters, indicative of a higher potential of carbohydrate biosynthesis in this environment. This is supported by previous observations of polysaccharides accumulating in environments exposed to higher light intensities and is in accordance with the observed decrease in polysaccharide concentration with depth [62]. Moreover, glycolysis was most abundant in surface waters, whereas gluconeogenesis, the glyoxylate shunt and oxidative phosphorylation were more common in >10,000 m waters. Recent studies showed that microorganisms tend to utilize some refractory organic carbon compounds (e.g., alkanes) in the deep-sea zones and labile carbohydrates in surface waters, respectively [2,63]. This indicates that more bacteria exploit labile carbohydrates via glycolysis at the surface. Higher relative abundance of the glyoxylate shunt may be an adaptive strategy for bacteria in >10,000 m waters to reduced carbon demand. Enriched oxidative phosphorylation suggests enhanced microbial mineralization activities in >10,000 m waters, as reported by significantly higher oxygen consumption rate in hadal sediments in the Challenger Deep using both chemical [64] and metagenomics [65] analyses. Among those complexes involved in oxidative phosphorylation, the *aa*_3_-type cytochrome *c* oxidase with low affinity for O_2_ had the lowest relative abundance at >10,000 m, while the bd-type cytochrome oxidase, with high affinity for O_2_ [66], had the highest relative abundance at this depth. This may be an adaptive strategy for bacteria at >10,000 m waters to maintain a high oxygen consumption rate.

Microbes in the deepest water (>10,000 m) also contained genes encoding an unusual form of nitrogen cycling. In previous studies, it was demonstrated that the α lineage of the ammonia-oxidizing archaeal (AOA) was distributed in the abyssal and hadal layers in the Mariana and Ogasawara Trenches [1,15]. Correspondingly, our study also found three AOA α lineage MAGs at ≥8000 m layers which possessed the archaeal *amoA* group E HAC (high ammonia concentration) (Figure 6). The α lineage of AOA prefer environments with higher ammonia concentrations than other lineages. However, the ammonia concentration was not high at all depths, ranging from 17.5 to 26.7 nM [2]. Interestingly, the relative abundance of nitrogen-fixing genes (*nifDKH*), the DNRA pathway and urea transporters in the deepest hadal zone (>10,000 m) were about four, two and three times higher than upper layers, respectively (Figure 2 and Figure 5). The enrichment of *nifDKH*, the DNRA pathway and transporters for urea uptake, could provide the additional ammonia demand required for the survival of this AOA lineage in the deepest ocean (>10,000 m).

## 5. Conclusions

In summary, this study has delineated the metagenome potential of the Mariana Trench to a fine scale and revealed different seawater layers harboring distinct microbial populations and metabolic potentials. Overall, the biodiversity increased with depth, and the genome size of MAGs at ≥4000 m layers was slightly larger compared to those at 0–2000 m. In addition, the deepest waters (>10,000 m) showed potential enhanced microbial activities, and an unusual form of nitrogen metabolism for ammonia-oxidizing archaea α lineage survival. This metagenomic study is limited in that predictions are based on genetic potential. Therefore, it is important in the future to test hypotheses generated in this and other metagenomics studies by performing detailed and focused studies that encompass process measurements, transcriptomics, proteomics and experiments on cultured microbes isolated from different depths. Nevertheless, this study provides profound implications for understanding how the functional roles of marine microbial communities vary with depth, and to our knowledge, is also the first to show the unique metabolic potential in the deepest waters on Earth.

## Supplementary Materials

The following are available online at https://www.mdpi.com/2076-2607/8/9/1309/s1, Figure S1: The genome size (A) and GC content (B) of MAGs. Figure S2: Phylum-level composition based on MAG genomes (A) and metagenomics reads (B) along the water column in the Challenger Deep. Figure S3: Distribution of major functional processes across different water depths in the Mariana Trench. Figure S4: Heatmap of distribution of genes encoding carbohydrate-active enzymes (CaZY) in the Mariana Trench. Figure S5: The relative abundances of genes involved in the microbial degradation pathways of peptides (A). The relative abundances of peptidase genes to that of CAZyme genes ratios (B). Figure S6: KEGG assignments to pathways related to hydrocarbon metabolism (A) and relative abundance of each step (B). Box plots of the sum of the relative abundances of alkanes (C) and aromatics (D) degradation genes. Box plots of the ratios of the relative abundance of alkanes to aromatics degradation genes (E). Table S1: Classification of MAG genomes based on their placement in the reference tree, relative evolutionary divergence, and ANI to reference genomes. Table S2: General component of the microbiome of MAGs and metagenomics reads. Table S3: Distribution of fractional percentage of processes in the 726 MAG genomes. Table S4: The abundance of MAGs in each sample they were extracted. Table S5: Distribution of CAZymes in 726 MAG MAG genomes.
