# Supplementary material for: Insights into the Vertical Stratification of Microbial Ecological Roles across the Deepest Seawater Column on Earth
Source: Microorganisms. 2020 Aug 27;8(9):1309. doi: 10.3390/microorganisms8091309 (PMC7565560; doi:10.3390/microorganisms8091309)
Supplement: Supplementary file 1 [file microorganisms-08-01309-s001.zip › Supplementary Material/Supplementary Material.docx]

Insights into the Vertical Stratification of Microbial Ecological Roles across the Deepest Seawater Column on Earth

Chun-Xu Xue ^1^, Jiwen Liu ^1,2^, David J. Lea-Smith ^3^, Gary Rowley ^3^, Heyu Lin ^4^, Yanfen Zheng ^1^, Xiao-Yu Zhu ^1^, Jinchang Liang ^1^, Waqar Ahmad ^1^, Jonathan D. Todd ^3^ and Xiao-Hua Zhang ^1,2,5,^*

Supplementary Figures


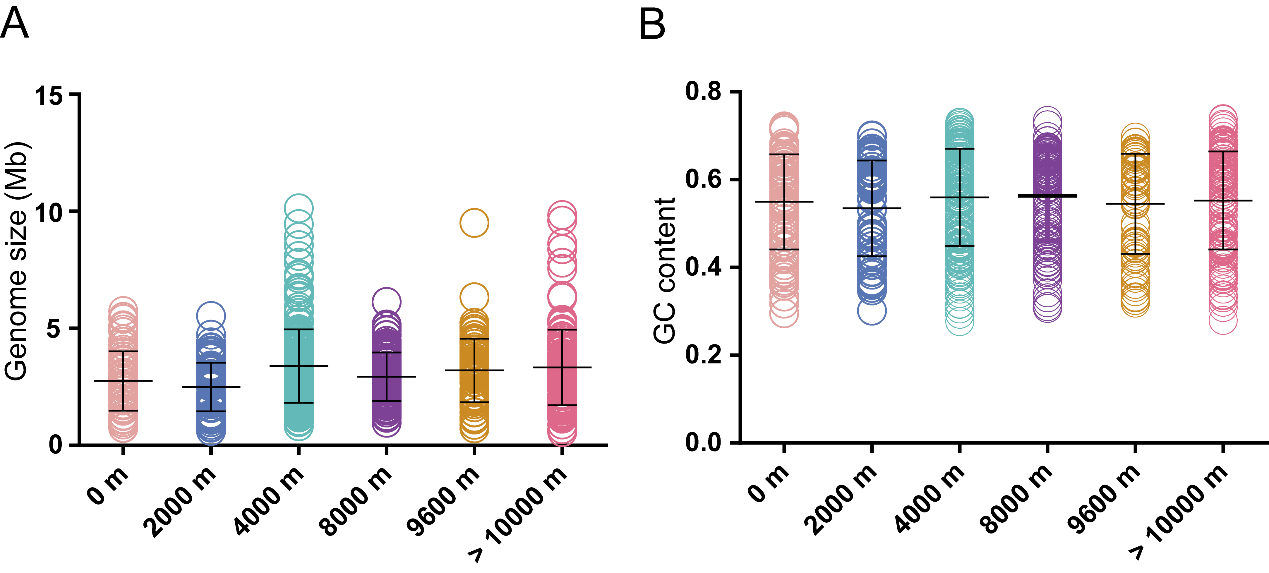


**Figure S1.** The genome size (**A**) and GC content (**B**) of MAGs. Horizontal black line indicates average genome size and GC content. Error bar indicates standard deviation.


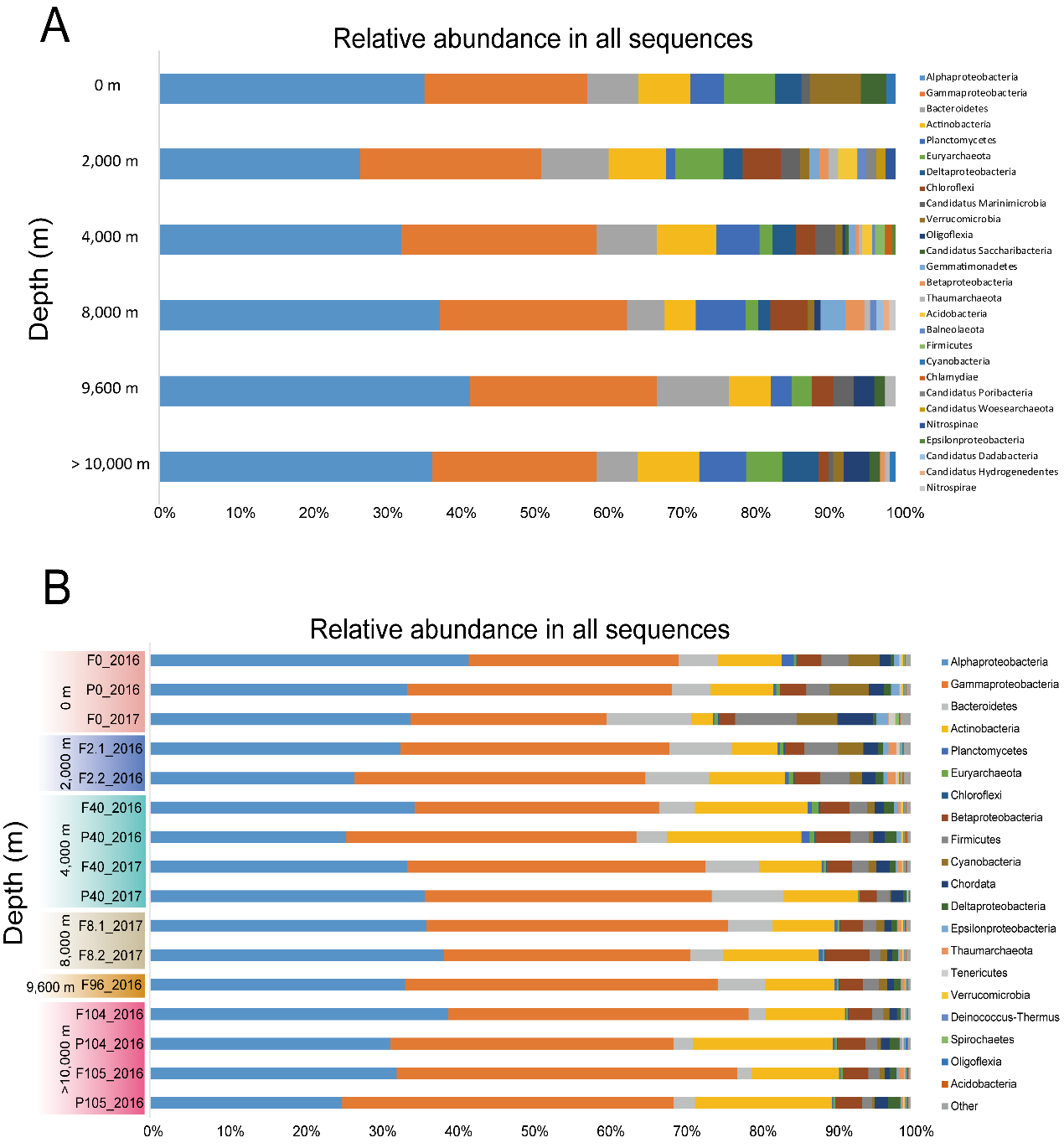


**Figure S2.** Phylum-level composition based on MAG genomes (**A**) and metagenomics reads (**B**) along the water column in the Challenger Deep. The phylum *Proteobacteria* was represented by its class-level classification. Only the relative abundances of the twenty most abundant bacterial phyla were shown. On the panel B, sample names in distinct color correspond to those collected in different water depth.


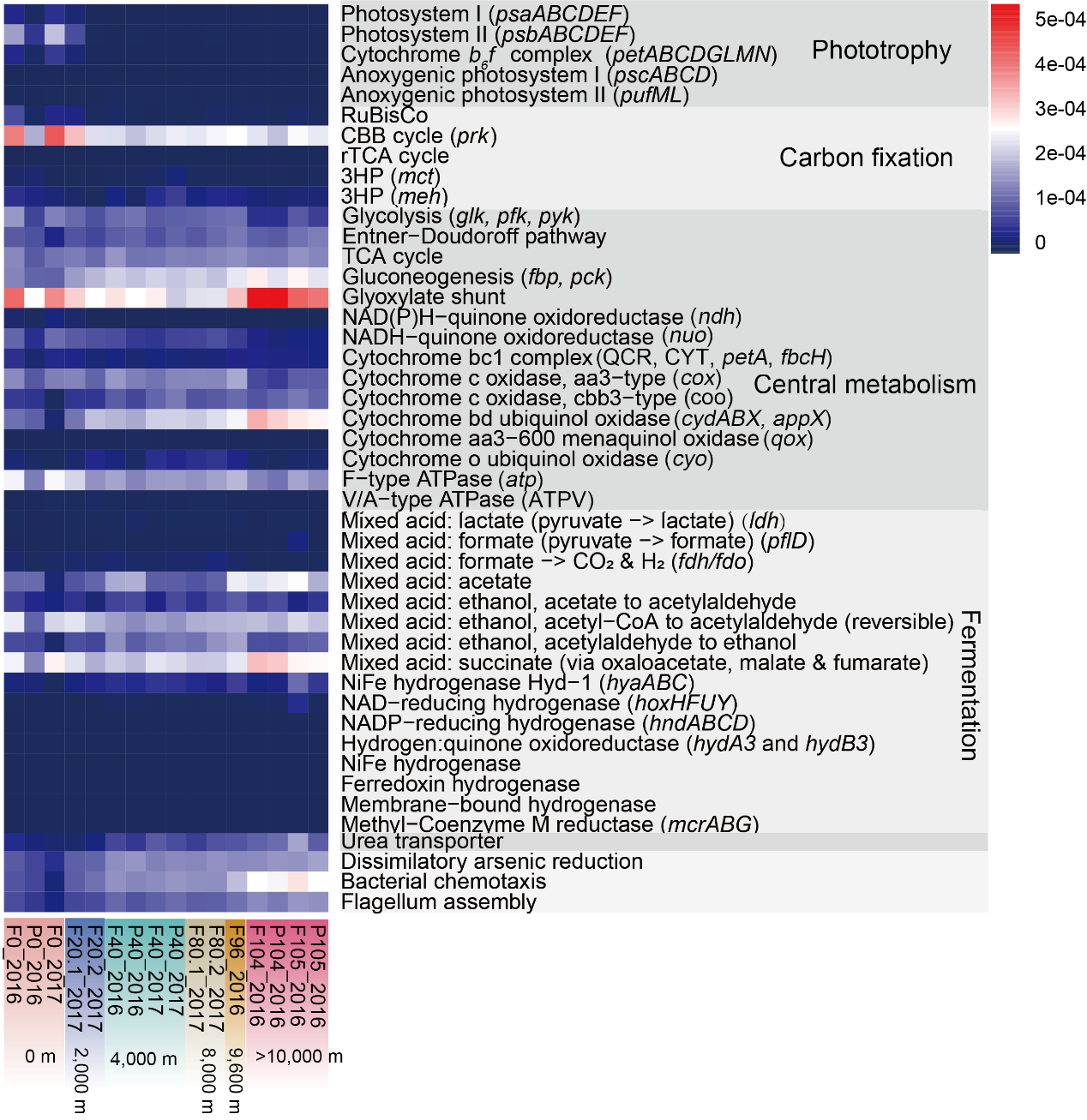


**Figure S3.** Distribution of major functional processes across different water depths in the Mariana Trench. A gradient from blue to red indicates pathway relative abundance across water layers. X-axis indicates the different water layers in several thousand meter increments (Sample names are defined by size fraction, sampling depth and sampling time, e.g., F0_2016 is the free living fraction at 0 m and sampling in 2016). The y-axis indicates the major selected functional processes involved in oxidative phosphorylation, carbon fixation, carbon degradation, hydrogen redox and other specific pathways.


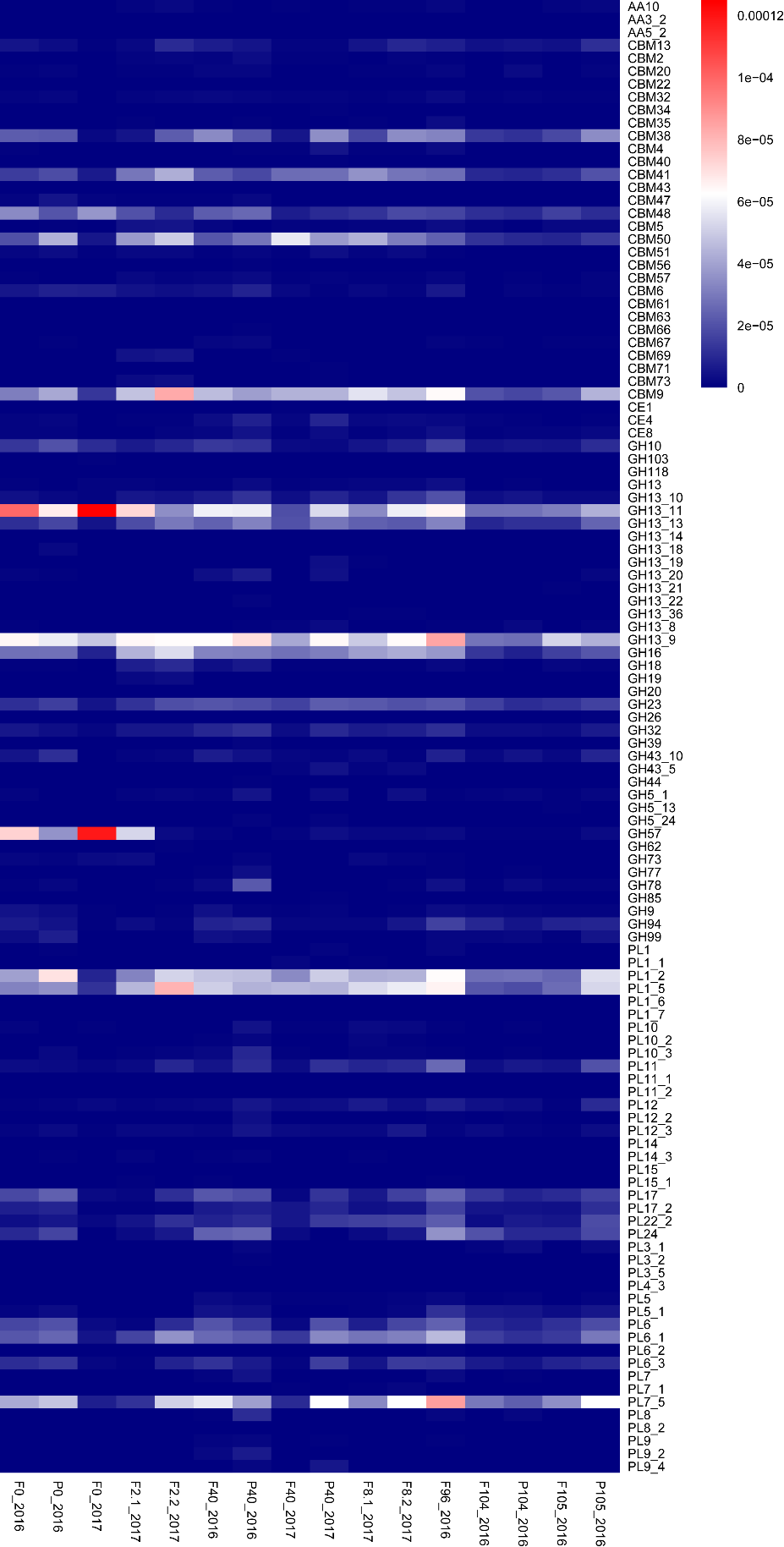


**Figure S4.** Heatmap of distribution of genes encoding carbohydrate-active enzymes (CaZY) in the Mariana Trench. A gradient from white to maroon indicates pathway relative abundance across water layers. X-axis indicates the different water layers in several thousand meters increment (Sample names are defined by size fraction, sampling depth and sampling time, e.g., F0_2016 is the free living fraction at 0 m and sampling in 2016), the y-axis the CAZymes identified. GH glycoside hydrolase, CBM carbohydrate-binding modules, PL polysaccharide lyase.


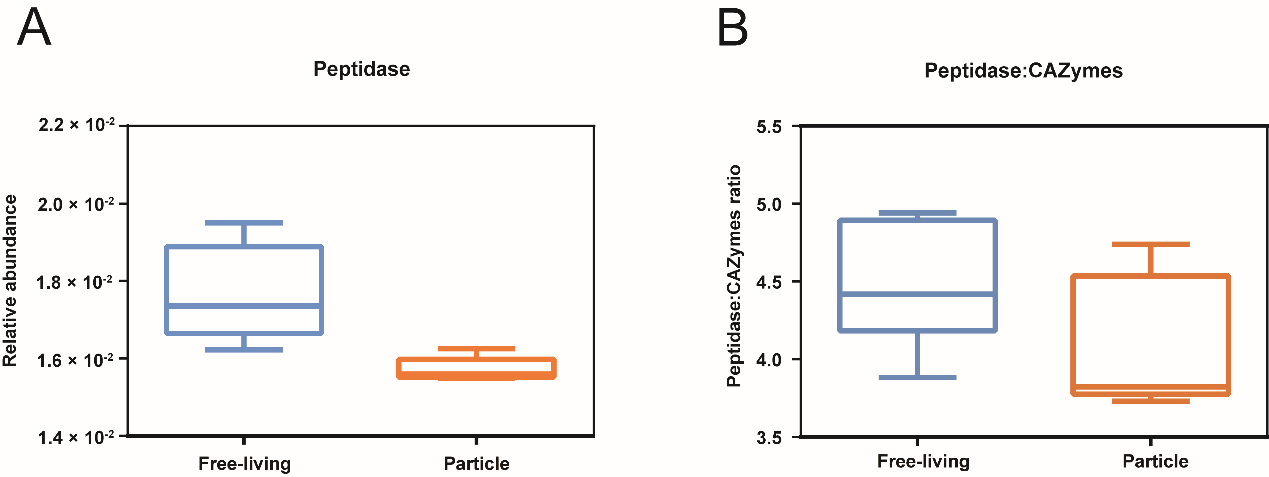


**Figure S5.** The relative abundances of genes involved in the microbial degradation pathways of peptides (A). The relative abundances of peptidase genes to that of CAZyme genes ratios (B).


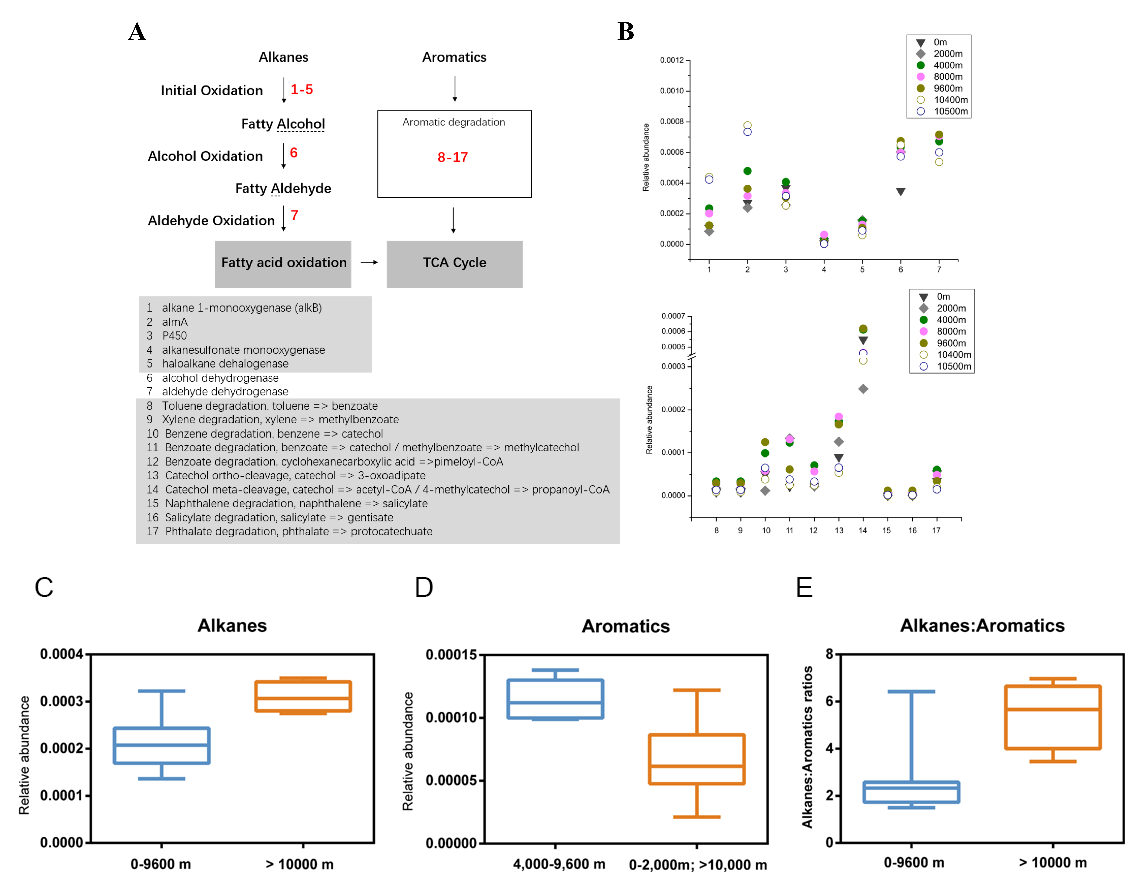


**Figure S6.** KEGG assignments to pathways related to hydrocarbon metabolism (A) and relative abundance of each step (B). Box plots of the sum of the relative abundances of alkanes (C) and aromatics (D) degradation genes. Box plots of the ratios of the relative abundance of alkanes to aromatics degradation genes (E).

Supplementary Table Legend

**Table S1.** Classification of MAG genomes based on their placement in the reference tree, relative evolutionary divergence, and ANI to reference genomes.

**Table S2.** General component of the microbiome of MAGs and metagenomics reads.

**Table S3.** Distribution of fractional percentage of processes in the 726 MAG genomes.

**Table S4.** The abundance of MAGs in each sample they were extracted.

**Table S5.** Distribution of CAZymes in 726 MAG MAG genomes.
