# Supplementary material for: Insights into the Vertical Stratification of Microbial Ecological Roles across the Deepest Seawater Column on Earth
Source: Microorganisms. 2020 Aug 27;8(9):1309. doi: 10.3390/microorganisms8091309 (PMC7565560; doi:10.3390/microorganisms8091309)
Supplement: Supplementary file 1 [file microorganisms-08-01309-s001.zip › Supplementary Material/TableS2.docx]

**Table S2** General component of the microbiome of MAGs and metagenomics reads

|  | 0 m | | | 2,000 m | | 4,000 m | | | | 8,000 m | | 9,600 m | >10,000 m | | | |
| --- | --- | --- | --- | --- | --- | --- | --- | --- | --- | --- | --- | --- | --- | --- | --- | --- |
|  | F0_2016 | P0_2016 | F0_2017 | F2.1_2016 | F2.2_2016 | F40_2016 | P40_2016 | F40_2017 | P40_2017 | F8.1_2017 | F8.2_2017 | F96_2016 | F104_2016 | P104_2016 | F105_2016 | P105_2016 |
| **Taxonomically assigned reads (%)** | 68.21 | 66.35 | 59.41 | 68.45 | 63.33 | 70.02 | 70.62 | 71.47 | 74.34 | 73.01 | 75.78 | 72.50 | 73.88 | 71.97 | 74.30 | 73.87 |
| *Bacteria* (%) | 96.70 | 95.70 | 90.71 | 95.08 | 95.94 | 97.23 | 97.54 | 96.75 | 97.79 | 97.97 | 98.34 | 98.18 | 95.49 | 98.01 | 96.77 | 98.62 |
| Archaea (%) | 0.35 | 0.48 | 0.59 | 1.46 | 1.66 | 1.21 | 0.62 | 0.91 | 0.16 | 0.73 | 0.67 | 0.57 | 0.55 | 0.50 | 0.98 | 0.47 |
| Eukaryota (%) | 1.30 | 1.84 | 4.21 | 1.81 | 1.67 | 1.14 | 1.40 | 1.67 | 1.57 | 0.90 | 0.63 | 0.90 | 0.96 | 1.07 | 0.62 | 1.68 |
| Viruses (%) | 1.29 | 1.63 | 3.67 | 1.17 | 0.28 | 0.10 | 0.11 | 0.34 | 0.30 | 0.18 | 0.15 | 0.14 | 0.08 | 0.14 | 0.12 | 0.14 |
| **MAGs** |  | | | | | | | | | | | | | | | |
| *Bacteria* (%) | 93.10 | | | 90.91 | | 97.77 | | | | 97.54 | | 95.77 | 87.59 | | | |
| Archaea (%)  Genome size (Mb)  GC content | 6.90  0.75-5.74  0.30-0.72 | | | 9.09  0.62-5.53  0.30-0.70 | | 2.23  0.82-10.14  0.28-0.73 | | | | 2.46  0.70-6.13  0.31-0.74 | | 4.23  0.74-9.50  0.32-0.70 | 12.41  0.57-9.84  0.28-0.74 | | | |
